# Recent Advances in the Application of Silver Nanoparticles for Enhancing Phototherapy Outcomes

**DOI:** 10.3390/ph18070970

**Published:** 2025-06-27

**Authors:** Rebeca M. Melo, Gabriela M. Albuquerque, Joalen P. Monte, Giovannia A. L. Pereira, Goreti Pereira

**Affiliations:** 1 Departamento de Química Fundamental, Universidade Federal de Pernambuco, Recife 50670-901, Braziljoalen.monte@ufpe.br (J.P.M.); 2 Departamento de Química & CESAM, Universidade de Aveiro, 3810-193 Aveiro, Portugal

**Keywords:** plasmonic nanoparticles, nanostructures, photothermal therapy, photodynamic therapy, photodynamic inactivation

## Abstract

The therapeutic use of silver nanoparticles (AgNPs) has been increasing, especially in phototherapy strategies. The plasmonic properties of AgNPs have contributed to their excellent results as phototherapeutic agents, namely for photodynamic therapy (PDT), photothermal therapy (PTT), and photodynamic inactivation of microorganisms. Moreover, the capacity of these nanostructures to release silver ions (Ag^+^) and enhance the production of reactive oxygen species (ROS) has been explored in combination with light to treat several diseases. Moreover, synthesis, functionalization, and conjugation strategies with targeting agents have been widely studied to optimize selectivity and maximize the therapeutic efficacy of these nanoplatforms. In this work, we reviewed the recent advancements (2019–2024) in the use of AgNPs for phototherapy applications, with an emphasis on evaluating therapeutic efficacy and specific targeting. According to the literature, in oncology, AgNPs have been predominately employed in PTT-based strategies, demonstrating significant tumor cell death and preservation of healthy tissues, in both *in vitro* and *in vivo* studies. Concurrently, AgNP-mediated PDT has emerged as a promising approach for the eradication of bacteria and fungi, particularly those commonly associated with antibiotic resistance. The compiled data indicate that AgNPs represent an innovative and effective therapeutic alternative, with a strong potential for clinical translation, in both cancer treatment and the management of hard-to-treat infections.

## 1. Introduction

Light-based therapeutic strategies have emerged as novel concepts with the potential to overcome issues related to traditional treatment approaches [1]. One of the biggest challenges in the medical field is cancer treatment. Chemo- and radiotherapy are the most widely used forms of cancer treatment. However, they are considered invasive methods, since they cause side effects throughout the treatment and in the long term [2]. Another emerging concern is bacterial resistance to antibiotics, which has been challenging for the treatment of infections, mainly in a hospital environment [3]. Therefore, therapies involving the local incidence of light, such as photothermal therapy (PTT) and photodynamic therapy (PDT), have emerged as promising and minimally invasive alternatives. These therapies have exhibited several advantages, such as a non-invasive nature, fewer side effects, and minimal drug resistance [2,3]. Furthermore, phototherapy has been associated with other therapeutic methods, such as chemotherapy, enhancing treatment efficacy and reducing side effects [4,5].

Despite the advantages of phototherapy, its efficiency is restricted by light penetration into biological tissue, where the depth of penetration is largely dependent on the light’s wavelength [6,7]. UV and visible light have lower tissue penetration due to absorption and scattering within the human body. On the other hand, near-infrared (NIR) light allows deeper penetration, presenting lower light absorption or scattering by biological tissues. This region can be divided into two: the first window (NIR-I, 700–950 nm), which allows a penetration up to 1–2 mm into the skin, and the second window (NIR-II, 1000–1350 nm), which enables light penetration of 2–3 mm into the tissue [7,8]. Moreover, long exposure times and intense light sources can result in phototoxicity. For instance, UV light can damage DNA and blue light induces cell death [8,9]. Thus, phototherapy agents activated by NIR light can enhance the application and efficacy of phototherapy methods [4,8].

In recent years, the design of advanced nanomaterials for phototherapy has grown significantly, to produce light-absorbing agents that are capable of converting light into thermal energy or enhancing the reduction–oxidation reactions at the desired site, with low invasiveness and high efficiency, often complementing traditional therapies [10]. Several nanoparticles (NPs) have been designed to overcome the limitations of traditional photosensitizers (PSs), including metallic nanostructures, semiconductor quantum dots, carbon dots, and upconversion NPs [11,12]. Among these nanomaterials, metallic nanostructures, such as gold (Au-) and silver nanoparticles (AgNPs), are promising nanostructures for phototherapy applications [12,13,14]. At the nanoscale, these materials exhibit localized surface plasmon resonance (LSPR), a phenomenon that significantly affects their thermal, optical, and electronic properties, making them attractive as photosensitizing agents (PAs) [13,14].

AgNPs have been used to convert light energy into thermal energy, generate reactive oxygen species (ROS), and act as antenna to enhance the efficiency of PSs [8,14]. Furthermore, the use of NPs in biomedical applications takes advantage of the enhanced permeability and retention (EPR) effect, a phenomenon that enables greater penetration and longer retention times in cancer cells compared to normal cells [15,16]. Moreover, the surface of AgNPs can be modified to extend their systemic circulation lifetime and promote selective bioaccumulation [8,17]. These features can increase the targeting of diseased cells, and enhance the accumulation of AgNPs at the desired site, allowing localized therapy [14,18].

Several published reviews have discussed the use of AgNPs in biomedicine [14], as antibacterial agents [19,20], dentistry applications [21,22], and cancer therapy [23]. However, to the best of our knowledge, none of these review articles have addressed the association between AgNPs and light for therapeutic purposes. Thus, this review summarizes the recent progress (2019–2024) on the use of AgNPs in phototherapy approaches, including PDT, PTT, and combined therapies, for treating cancer and inactivating microorganisms.

## 2. Silver Nanoparticles

In recent years, plasmonic nanoparticles have gained considerable attention owing to their optical and physicochemical properties, allowing their applicability in different areas. These NPs have an active surface and a high surface-area-to-volume ratio, enabling their functionalization with several (bio)molecules and enhancing their interaction capabilities [24]. In addition, metallic nanostructures exhibit an LSPR band, resulting from their interaction with light, varying from the visible to the NIR region. Among these nanostructures, AgNPs offer several advantages over other metals, such as simple synthesis methodologies, high control over size and morphology, tunable absorption region (from UV to NIR) as a function of its shape, excellent electrical and thermal conductivities, high reflectance, effective light-to-heat conversion, good cost–benefit, and lower toxicity compared to other metals [12,24].

### 2.1. AgNPs’ Preparation and Properties

AgNPs can be prepared using physical (like laser ablation), chemical (e.g., chemical reduction, sonochemical, microwave, and colloidal synthesis), and biological methods (using microorganisms) [25,26]. The choice of the synthetic method and the experimental parameters used directly influence the size and morphology of the AgNPs, which can be spherical, prismatic, cubic, rod- or platelet-shaped [24,27].

Chemical reduction is the most commonly used synthetic route, requiring a silver precursor, stabilizing agent, and reducing agent [25]. Moreover, several strategies have been applied to control the nanostructure shape, including the use of hydrogen peroxide, light irradiation, seed-mediated synthesis, and shape-directing molecules [24,28,29].

**Figure 1:**
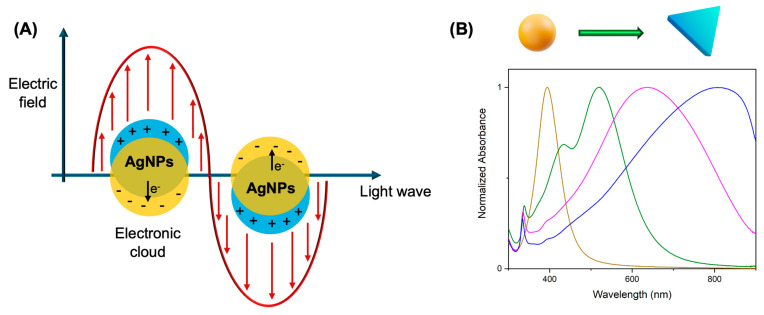
(**A**) Schematic representation of the localized surface plasmon resonance phenomenon of AgNPs when interacting with light. (**B**) Correlation between the absorbance spectra of AgNPs and their shape, where spherical AgNPs typically exhibit an absorption band around 400 nm, while prismatic AgNPs show broader absorption ranging from 550 to 1000 nm.

AgNPs exhibit LSPR, a phenomenon arising from the collective oscillation of conduction band electrons in response to incident light. When the AgNPs are exposed to an electromagnetic field, the electrons in the metals’ conduction band oscillate collectively and in resonance with the light frequency, generating the LSPR at the AgNPs’ surface. This effect significantly influences the optical properties of AgNPs and modulates their interaction with the surrounding environment. For NPs with sizes smaller than the light wavelength, the surface plasmon oscillation originates a dipole moment, since the electrons move coherently in opposition to the external electric field (Figure 1A) [12,30].

The LSPR of these nanostructures can be tuned by adjusting their shape and size, and consequently, the color of the colloidal suspension also changes. Spherical AgNPs’ colloidal suspensions exhibit a yellow color with a single absorption band at approximately 400 nm, corresponding to a unique dipole oscillation mode of the conduction electrons. On the other hand, asymmetrical nanostructures, such as prismatic AgNPs, exhibit multiple plasmon resonance modes with a maximum at 550–1000 nm, resulting in a broader absorption band and colloidal suspensions with colors ranging from violet to blue [12,24,27] (Figure 1B). Therefore, AgNPs can ensure more effective absorption and greater local heating for treatments that use light from the visible to the NIR [12].

### 2.2. Therapeutical Applications of AgNPs

AgNPs have demonstrated great potential as therapeutic tools for cancer therapy, bacterial infections, tissue engineering, and wound healing [31]. These NPs have already been approved by the Food and Drug Administration (FDA) as antibacterial agents for clinical use [32].

The AgNPs’ antibacterial mechanism has been primarily attributed to the release of Ag^+^ and their ability to penetrate bacterial cell membranes. The generation of Ag^+^ has been associated with the oxidative degradation of the AgNPs in the surrounding medium. These ions can interact with cellular proteins and DNA, alter the bacteria’s metabolism, and disrupt the cell membrane, leading to bacteria death [33,34]. Additionally, AgNPs can induce the spawning of ROS or impair the bacteria’s ROS protection mechanisms, both of which cause the cells’ oxidative stress and apoptosis [33]. Thus, AgNPs have been incorporated into or associated with several biomaterials to prevent bacterial infections, in applications such as wound healing [35] and tissue regeneration [36].

The therapeutic efficiency of AgNPs is affected by several factors, such as size, shape, and surface ligands. Smaller AgNPs have been appointed as having greater reactivity compared to larger ones, due to their higher surface area-to-volume ratio and enhanced cellular uptake. The size control of AgNPs can be achieved by adjusting parameters such as pH and reagents’ molar ratios used during synthesis [33,34]. For instance, Pucelik et al. [37] investigated the biological activity of spherical AgNPs coated either with trisodium citrate (TSC) or *N,N,N*-trimethyl-(11-mercaptoundecyl) ammonium chloride (TMA). These authors prepared two negatively charged AgNPs-TSC with diameters of 5.5 and 10.7 nm, and two positively charged AgNPs-TMA measuring around 2.8 and 11.0 nm. The ultra-small AgNPs-TMA presented a greater cellular uptake in both Gram-positive (*Staphylococcus aureus*) and Gram-negative (*Escherichia coli*) bacteria, as well as in mammalian cells. Furthermore, the TSC-coated AgNPs exhibited stronger binding affinity to Gram-positive bacteria, while the positively charged AgNPs-TMA were more efficiently internalized by Gram-negative bacteria. These authors also concluded that although smaller AgNPs-TMA presented higher overall accumulation in mammalian cells, the larger AgNPs-TMA offered a better balance between cellular uptake, specific interaction with cancer cells, and reduced toxicity to healthy cells [37].

In another work, Gibala et al. [38] prepared a series of AgNPs with similar sizes but capped with different stabilizing agents. The absorption spectra confirmed the successful formation of spherical AgNPs, which was further verified by transmission electron microscopy (TEM), revealing average diameters ranging from 10 to 17 nm. AgNPs stabilized with cysteamine, cysteine, lysine, and arginine exhibited a positive surface charge. In contrast, AgNPs capped with TSC, gallic acid, epigallocatechin gallate, tannic acid, caffeine, hydroxylamine hydrochloride, and sodium hexametaphosphate were negatively charged. The results showed that the negatively charged AgNPs were more biocidal against *E. coli*, with the one capped with lysine being the most effective. Meanwhile, tannic acid- and caffeine-capped AgNPs demonstrated higher activity against *S. aureus*. Additionally, studies with *Candida albicans* showed that AgNPs stabilized with lysine or hydroxylamine hydrochloride exhibited the highest efficiency in inactivating this pathogen. Thus, the authors concluded that the effectiveness of AgNPs cannot be determined solely by their surface charge, as their activity also depends on the biological organism under study. Moreover, the AgNPs’ ability to convert light into heat upon excitation has been explored for phototherapeutic applications [38].

### 2.3. Role of AgNPs in Phototherapy

AgNPs are promising nanostructures for phototherapy due to their LSPR feature. Upon light irradiation, the collective oscillation of surface free electrons generates an intense electromagnetic field around the NPs, enhancing light absorption and scattering [12].

**Figure 2:**
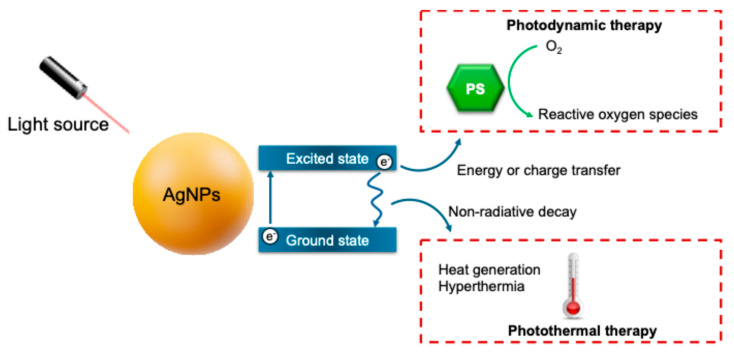
Representative mechanisms by which AgNPs enhance phototherapy: in PDT, through transfer energy to the photosensitizer (PS), boosting ROS production; and in PTT, via light-to-heat conversion leading to localized hyperthermia.

AgNPs can be conjugated with PSs to enhance PDT outcomes. Due to the LSPR properties, AgNPs function as nanoscale antennas, amplifying the local electromagnetic field and thereby improving the light absorption efficiency of nearby PS molecules (Figure 2). However, for optimal treatment enhancement, the absorption band of the AgNPs used should be tuned to be in the same wavelength region as the incident laser. Moreover, there must be a spectral overlap between the extinction profile of the AgNPs and the absorption spectrum of the PSs. Thus, it is crucial to select the AgNPs’ optical properties according to the PSs’ absorption spectra [11]. For example, Rodrigues et al. [39] produced several prismatic AgNPs with LSPR maxima at different spectral regions, in order to obtain optimal overlap with the absorption spectrum of methylene blue. Their results showed that the prismatic AgNPs exhibiting an LSPR band near 621 nm demonstrated the most significant enhancement in PDT efficiency when combined with methylene blue. In another example, Mota et al. [40] showed that the distance between the AgNPs and PSs can also influence the PDT effect. The AgNPs-PS distance was controlled by thin layers of Pluronic copolymers. Pluronic-capped AgNPs were obtained in a spherical shape (λ_abs_ = 420 nm), and three PSs (curcumin, methylene blue, and eosin Y) were tested. The results demonstrated that the fluorescence emission and the production of ROS by the PSs were enhanced in the presence of the AgNPs-Pluronic, but not with bare AgNPs. The polymer coating provided a separation of 9.6 to 15.5 nm between the AgNP and the PS, which is under the optimal distance (5–20 nm) and crucial to promote enhancement of the PSs’ photophysical properties [40].

On the other hand, the electron relaxation in AgNPs primarily occurs through non-radiative decay pathways, converting a significant portion of absorbed light into heat and increasing the local temperature (Figure 2). This hyperthermia effect has been explored in PTT to accomplish the ablation of cancer cells [41]. To improve the efficiency of these treatments, it is necessary to synchronize the absorption band of the AgNP with the incoming light wavelength. In the LSPR band, the conversion yield of electromagnetic energy to heat is maximum [42].

Additionally, the development of AgNPs with the plasmonic resonance band in the NIR region enhanced the interest in these nanostructures, as NIR light enables deeper tissue penetration with minimal damage [23,42].

**Figure 3:**
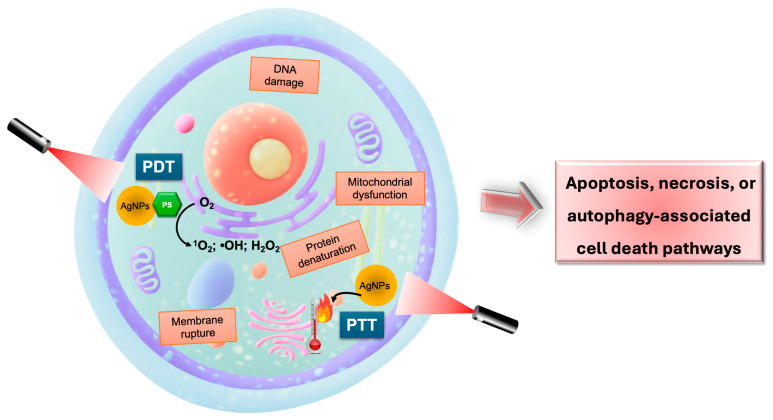
Schematic representation of possible cellular damages triggered by AgNP-based phototherapy.

The mechanisms underlying PDT and PTT following cellular uptake of AgNPs are complex, involving multiple phenomena that can ultimately lead to cell death (Figure 3). In PDT, once the AgNP-PS system is internalized by the cells and activated by light irradiation, the production of ROS initiates a cascade of reactions that lead to the cell’s oxidative stress and, consequently, their death. ROS can damage critical biomolecules, such as proteins, DNA, and lipids, resulting in apoptosis or necrosis. In addition, these oxidative reactions can lead to mitochondria dysfunction, promote apoptosis, or damage organelles like lysosomes and the endoplasmic reticulum, inducing autophagy [1,16]. Moreover, oxygen consumption inside the cells to yield ROS creates an hypoxia state that decreases the cell’s functions [16,43].

In PTT, light irradiation of AgNP-treated cells provokes a localized temperature rise in the tissues leading to cell apoptosis or necrosis. When this temperature surpasses 39 °C, denaturation and aggregation of proteins and other biomolecules may occur. As temperature increases further, cells attempt to counteract thermal damage by producing heat shock proteins. At 43–45 °C, accelerated biochemical reactions and elevated ROS levels further exacerbate cellular stress, leading to cell damage [1,44].

### 2.4. Toxicity of AgNPs

The toxicity of AgNPs is a significant concern for their use in humans. Their potential health impact is related to toxicological effects such as oxidative stress, inflammation, and cellular toxicity. However, these toxicological implications can be mitigated by modulating the physicochemical properties of AgNPs, including their dosage, surface coating, charge, size, and shape. Thus, the therapeutic application of AgNPs required a balanced and well-informed approach, with careful consideration of their intrinsic characteristics and dose-dependent effects [45,46].

*In vivo* studies using animal models have shown that smaller AgNPs exhibit greater toxicity compared to larger ones due to their higher surface area and enhanced ability to penetrate biological membranes [47]. Additionally, increasing AgNP dosage decreases cell viability due to excessive Ag^+^ release, ROS yield, and potential damage to DNA, lipids, and cellular proteins [45,48]. On the other hand, Oćwieja et al. (2024) [49] investigated the toxicity of cysteine-stabilized AgNPs, both positively charged (AgNPs-Cys(+)) and negatively charged (AgNPs-Cys(−)). The study concluded that positively charged AgNPs exhibit higher toxicity, reducing lymphocyte viability and causing more pronounced genotoxicity and membrane disruption, compared to their negatively charged counterparts. The comparison of the cytotoxic effects of AgNPs with different surface charges—positive (ε-poly-L-lysine, PLL), neutral (polyvinylpyrrolidone, PVP), and negative (bis(2-ethylhexyl) sulfosuccinate sodium, AOT)—was evaluated by Vuković et al. (2020) [50]. This study found that positively charged AgNPs induce a more pronounced cytotoxic effect. Specifically, a concentration of 5 mg Ag.L^−1^ of PLL-AgNP resulted in 61.5% apoptosis in human peripheral blood mononuclear cells (hPBMCs) after 1 h of exposure, whereas PVP-AgNP and AOT-AgNP exhibited apoptotic rates of 48.5% and 23.5%, respectively, after 3 h of treatment.

Some research groups have also been studying AgNPs’ excretion and accumulation during long periods of exposure. Recordati et al. [51] studied the effect of 4-week oral exposure to 0.25 and 1 mg Ag.Kg^−1^ in mice. For this, spherical AgNPs stabilized with citrate and with a diameter of 10 nm were used. The authors observed that the accumulation of AgNPs in organs was dose dependent, and the mice treated with Ag salts showed higher ratios of Ag accumulation. Additionally, even after a period of recovery (28 days after treatment), a significant level of Ag was still detected in the brain and testis. In another study, Rosário et al. [52] exposed mice to AgNPs-PVP (with 5 and 50 nm), using the dosage of 1 and 3 mg Ag.Kg^−1^ during 28 days. These authors observed the presence of Ag in all organs analyzed, regardless of AgNP size. The major part of the Ag^+^ was excreted by the urinary tract, and the highest levels were observed for animals treated with Ag salt (AgNO_3_) and AgNPs-PVP with 50 nm, while in feces, the highest concentrations of Ag^+^ were found in mice administrated with AgNO_3_ and AgNPs with 5 nm. Furthermore, these authors showed that the AgNPs’ effects were size dependent, with the smaller AgNPs presenting an extensive accumulation in several organs, like the lung, spleen, kidney, and liver.

Given these studies, AgNP toxicity needs to be minimized through strategic design that carefully considers nanoparticle size, surface charge, and dosage, ensuring a balance between therapeutic efficacy and safety. Therefore, controlled manipulation of the physicochemical properties of AgNPs is essential to reduce health risks and enable their safe use in biomedical applications [53].

AgNPs are already being commercialized, mainly as nanoformulations for bacterial or viral infections, and the concerns about their toxicity still impact their transition to clinical trials. Key challenges include a limited understanding of their biodistribution, long-term accumulation, and endogenous behavior in humans. As a result, their use has been largely restricted to topical applications. Several AgNPs reached clinical trials; however, the majority of them failed to be translated to clinical uses [54]. Among the major obstacles to the clinical translation of these NPs is the lack of knowledge regarding their mechanisms of action, which makes it challenging to determine appropriate dosing and intervals of administration. In many cases, the clinical trials involving AgNPs showed that these systems are not therapeutically superior to commonly used pharmaceuticals. However, they have shown acceptable safety profiles for use in humans [15,54].

## 3. Application of Silver Nanoparticles in Phototherapy

In the past, AgNPs were identified as promising antimicrobial agents, with the potential to overcome bacterial antibiotic resistance. Therefore, the major applications of AgNPs in therapy remain focused on this area. Nevertheless, AgNPs are increasingly being employed to address other health issues. Recent studies have demonstrated that combining AgNPs with light can improve treatment efficacy, potentially leading to more effective strategies for combating several diseases and improving patient outcomes. Notably, AgNPs have been associated with light in the development of novel approaches to treat bacterial infections, fungal infections, and cancer, both *in vitro* and *in vivo*.

**Figure 4:**
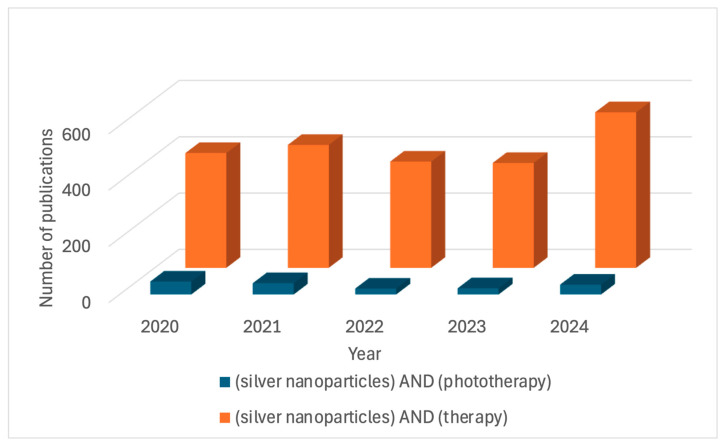
Results from the search in the Pubmed^®^ database using the keywords “Silver nanoparticles” combined with “Therapy” (orange), and “Phototherapy” (blue), for the period 2020–2024.

According to the Pubmed^®^ database, the number of papers that described the use of AgNPs for therapy has been increasing, counting more than 2000 articles in the last five years (Figure 4). When “Phototherapy” and “Silver nanoparticles” were used as the keywords, this number was reduced to 164 works in the last five years (Figure 4). We focus our review on these results, including the ones that use AgNPs for therapy under light irradiation, and excluding the ones that use nanoparticles containing or doped with silver ions.

### 3.1. Photodynamic Therapy of Cancer Cells

PDT is a non-invasive therapeutic method with significant therapeutic efficiency and negligible side effects. It consists of the selective damage of pathological cells and tissues by activating a light-absorbing molecule, known as a PS with light irradiation in the presence of molecular oxygen (^3^O_2_). When the PS is excited at a specific wavelength, it generates ROS or singlet oxygen molecules (^1^O_2_), which damage cancer cells by apoptosis [55,56]. An ideal PS selectively targets cancer cells with minimal uptake by healthy cells [57]. Among the most commonly used PSs for PDT, it is worth highlighting porphyrin, curcumin (Cur), 5-aminolevulinic acid (5-ALA), and its derivatives [55,57].

AgNPs have been combined with PSs to enhance their photophysical properties and achieve better PDT results. Moreover, together with the production of ROS, these NPs can release Ag^+^, further promoting cell death [58,59]. Furthermore, the use of NIR light sources in PDT has been growing because of their deeper tissue penetration and lower phototoxicity, compared to UV or visible light [57,58]. Thus, AgNPs with different shapes can increase absorption in this region. Overall, for cancer treatment based on PDT approaches, AgNPs have been synthetized in a spherical shape and were mainly associated with several PSs, such as phthalocyanines (Pcs) [60,61,62], porphyrins (Pps) [58], and Cur [63] (Table 1).

The use of PDT for breast cancer has been most explored with PSs containing spherical AgNPs. These AgNPs have been linked to zinc phthalocyanines (ZnPcs), an NIR-emitting xanthene derivative, or 5-ALA, without association with a specific cancer-targeting molecule. To achieve a PS with NIR light emission, Liu et al. [59] prepared an AgNP–carbon dot nanocomposite (AgNP-CD) coated with a 2,3-dihydro-1H-xanthene-6-ols derivative (CyOH), enabling a system for fluorescence imaging and efficient tumor treatment. The AgNPs-CD-CyOH system generated more ^1^O_2_ than AgNP-CD, under irradiation with a 660 nm laser (60 mW.cm^−2^) for 5 min. The therapeutic efficiency of this system was evaluated in breast cancer cells (4T1 cells) by incubating the cells for 8 h, and then submitting them to NIR irradiation. This system exhibited negligible cytotoxicity in the absence of laser irradiation. However, after 5 min of irradiation (660 nm laser), 10% cell viability was observed. *In vivo* tests were performed on mice infected with 4T1 cells. After PDT, the animals treated with AgNPs-CD-CyOH showed apparent tumor shrinkage, which was confirmed 20 days post-treatment by hematoxylin and eosin (H&E) staining [59].

**Figure 5:**
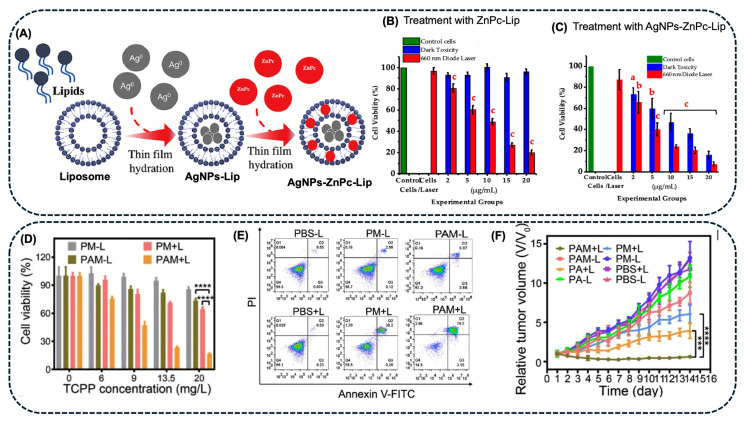
(**A**) Schematic representation of the formation of the AgNPs and ZnPc liposomal (Lip) formulation. MCF-7 cell viability in the presence of (**B**) ZnPc-Lip and (**C**) AgNPs-ZnPc-Lip, without and with a 660 nm laser irradiation. ^a^ *p* < 0.05, ^b^ *p* < 0.01, and ^c^ *p* < 0.001. Reprinted from Chota et al. [64], Copyright (2024), with permission from Elsevier. (**D**) Viability of CT26 cells to porphyrinic network in a neutrophil membrane (PM) and porphyrinic network-AgNPs-neutrophil membrane (PAM) in the absence (−L) and presence (+L) of light (660 nm, 0.22 W.cm^−2^). (**E**) Apoptosis analysis of CT26 cells treated with PBS, PM, and PAM, with or without light. (**F**) Relative volume of CT26 tumors of infected mice, submitted to different treatments: PBS, AgNPs-PCN (PA), PM, and PAM, in the absence and presence of light irradiation. *** *p* < 0.001, **** *p* < 0.0001. Reprinted from Zhang et al. [58], Copyright (2020), with permission from Elsevier.

In a recent study, Chota et al. [64] incorporated AgNPs and a ZnPc into a liposomal formulation for PDT of breast cancer (MCF-7). The AgNPs were prepared using a root extract of *Dicoma anomala*, yielding spherical NPs with an average size of 36 ± 7 nm and maximum absorption at 425 nm. These AgNPs were subsequently incorporated into liposomes (Lip) along with zinc phthalocyanine tetrasulfonate, using the thin-film hydration methodology (Figure 5A). The irradiation of AgNPs-ZnPc-Lip and ZnPc-Lip with a 660 nm laser showed that the quantity of ROS generated was higher for the formulation containing only the PS (ZnPc-Lip), achieving a singlet oxygen quantum yield of 0.11 and 0.06, respectively. The treatment of MCF-7 cells with AgNPs, AgNPs-Lip, ZnPc-Lip + laser, and AgNPs-ZnPc-Lip + laser showed cellular toxicity. However, the PDT treatment of MCF-7 cells with ZnPc-Lip and AgNPs-ZnPc-Lip, irradiated with a 660 nm laser, gave similar results (Figure 5B,C) [64].

To treat colon cancer, some authors have studied the association between AgNPs and Pp or Cur. Zhang and collaborators [58] developed a nanoplatform consisting of a porphyrinic porous coordination network (PCN) containing AgNPs coated with a neutrophil membrane (NM), achieving the AgNPs-PCN-NM system to be used as a PS. NM was intended to target cells and increase PS accumulation at the tumor site. During laser irradiation (660 nm, 0.22 W.cm^−2^), ^1^O_2_ was generated, creating a highly oxidative environment and releasing Ag^+^ ions to induce cell death through oxidative stress. The degradation of AgNPs was accompanied by a decrease in the absorption maximum at 420 nm and by inductively coupled plasma mass spectrometry (ICP-MS). *In vitro* assays were performed by incubating CT26 cells with AgNPs-PCN-NM (10 μg.mL^−^^1^) for up to 4 h and irradiating them with the laser for 5 min, resulting in a decrease in cell viability to 25% and 79.2% of apoptosis, in the presence of light (Figure 5D,E). For *in vivo* tests, AgNPs-PCN-NMs were systematically administered to mice, demonstrating excellent antitumor activity without evident side effects (Figure 5F) [58].

To enhance the quantum yield of singlet oxygen (Φ^1^O_2_), Freitas et al. [63] incorporated Cur combined with spherical AgNPs (*λ*_abs_ = 410 nm) into a hydrogel composed of chitosan (Chi) and chondroitin sulfate (CS). The Φ^1^O_2_ of the AgNPs-Cur conjugate was 14 times higher than that of Cur alone. Caco-2 human colon cancer cells were incubated with the AgNPs-Cur-Chi-CS for 24 h at 37 °C. The results demonstrated that all the systems tested did not cause cell death in the absence of light, nor did the hydrogel without AgNPs (Cur-Chi-CS) under light exposure, across a concentration range from 10.0 to 1000.0 μg.mL^−1^. In contrast, AgNPs-Cur-Chi-CS at 250.0 μg.mL^−1^ exhibited significant phototoxicity after PDT, resulting in less than 20% cell viability [63].

**Table 1:** Summary of recent works from the last 5 years, combining AgNPs and light irradiation to treat cancer treatment using PDT or PTT approaches.

Therapy Approach	Cancer Cells	System	Phototherapy Details	Results	Refs.
PDT	Breast Cancer	AgNPs-ZnPc	680 nm (400 mW.cm^−2^), 5 min	The attachment of AgNPs to ZnPc enhanced ROS production. AgNPs-ZnPc conjugates (80 μg.mL^−1^) had a lower PDT effect than ZnPc alone on MCF-7 cells.	[60]
		AgNPs-CD-CyOH	660 nm, 5 min	Cell viability significantly decreased (~10%) in the presence of AgNPs-CD-CyOH conjugates under light irradiation. Fluorescent microscopy experiments showed that AgNPs-CD-CyOH accumulated in the mitochondria of 4T1 cells.	[59]
		AgNPs-ZnPc	670 nm (2.3 W) *	The conjugation of AgNPs with ZnPc increased ROS generation under light irradiation. Although PDT studies showed changes in MCF-7 cellular morphology 24 h post-treatment, these conjugates exhibited better results in sonotherapy.	[61]
		AgNPs-PPA-5-ALA	635 nm (0.5 W.cm^−2^), 10 min	AgNPs-PPA-5-ALA (100 µmol.L^−1^) effectively killed 4T1 cells, under laser irradiation, *in vitro*. The tumors of mice treated with PDT using this system reduced tumor progression compared to the control groups.	[65]
		AgNPs-ZnPc	660 nm (10 J.cm^−2^) *	AgNPs and a ZnPc were encapsulated into a liposome (Lip) and used in PDT with MCF-7 cells. The *in vitro* studies showed a similar cytotoxicity of ZnPc-Lip and AgNPs-ZnPc-Lip, under irradiation with a 660 nm laser.	[64]
	Melanoma	AgNPs-ZnPc-FA	674 nm (9 mW.cm^−2^) *	The AgNPs-ZnPc-FA (2.5 µmol.L^−1^) loaded into mesoporous silica achieved approximately 92% cell death post-PDT procedure. Furthermore, the presence of FA enhanced the PDT efficacy against A357 cells.	[62]
	Cervical Cancer	AgNPs-AIE	White light (40 mW.cm^−2^), 10 min	AgNPs-AIE (10 μmol.mL^−1^) showed greater cellular uptake, compared to the AIE PS, and a high PDT efficiency, achieving around 85% cell death in HeLa cells.	[66]
	Colon Cancer	AgNPs-PCN	660 nm (0.22 mW.cm^−2^), 5 min	PDT treatment with AgNPs-PCN induced 79% apoptosis in CT26 cells *in vitro*. *In vivo* studies showed that the conjugate possessed excellent tumor-targeting capability, ROS yield, and antitumor activity.	[58]
		AgNPs-Cur	Blue light (25 mW.cm^−2^), 20 min	The conjugation of Cur with AgNPs enhanced its bioavailability. PDT with AgNP-Cur hydrogels (9.2 μg.mL^−1^) resulted in greater Caco-2 cell death.	[63]
	Lung Cancer	AgNPs-Phe	660 nm (10 J.cm^−2^) *	The PDT treatment with AgNPs (6.75 μg.mL^−1^) in combination with the PS pheophorbide a (Phe, 1.05 μmol.L^−1^) resulted in a significant cytotoxicity of A549 lung cancer cells.	[67]
PTT	Breast Cancer	AgNPs-PDA	808 nm (1 W.cm^−2^), 5 min	Prismatic AgNPs-PDA (10 μg.mL^−1^) achieved η = 41.9% with 5 min of irradiation. This system showed high PTT activity both *in vitro* and *in vivo*, irradiating 4T1 cells.	[68]
		AgNPs-HSA-DOX	808 nm (3 mW.cm^−2^), 5 min	After 15 min of irradiation, AgNPs-HSA presented a η of around 30%. PTT studies showed a 92.2% reduction in HS578T cell viability, in the presence of AgNPs-HSA-DOX (6.5 μmol.mL^−1^ of DOX), 6 h post-treatment.	[69]
		AgNPs-UCNPs-DOX	980 nm (0.45 W.cm^−2^), 3 min	The heating rate of AgNP-UCNPs increased under NIR irradiation. Systems containing AgNPs exhibited cytotoxicity, even in fibroblasts. Mice with induced breast cancer (Sk-Br-3 cells), after PTT with AgNPs-UCNPs-DOX (800 µg.mL^−1^), showed areas of necrosis and a reduced tumor volume compared to the controls.	[70]
	Triple-Negative Breast Cancer (TNBC)	AgNPs-FA-QRC	800 nm (1.5 W.cm^−2^), 5 min	AgNPs-FA-QRC, with a pyramidal shape, combined with NIR irradiation showed higher toxicity in MDA-MB-231 cells, presenting great antitumor activity in both *in vitro* and *in vivo* studies.	[71]
		AgNPs-PVP	970 nm (3 W), 70 s	Prismatic AgNPs-PVP (12.5 µg.mL^−1^), under NIR irradiation, exhibited greater cytotoxicity to MDA-MB-231 (TNBC) cells compared to MCF-10A (non-malignant) cells, decreasing the cell viability of TNBC to 15% and MCF-10A to 85%.	[72]
		AgNPs-Chi-DOX	808 nm (2 W.cm^−2^), 180 s	Prismatic AgNPs-Chi (10 μg.ml^−1^) showed η = 50.4%, under 10 min of NIR irradiation. Combined treatment with AgNPs-Chi-DOX (5 μg.ml^−1^) and 2 min of irradiation showed the best therapeutic results with MDA-MB-231 cells.	[73]
		AgNPs-PVA	808 nm (1.0 W.cm^−2^), 7 min	Prismatic AgNPs-PVA presented η = 30.4%. PTT with AgNPs-PVA (140 μg∙mL^−1^) in mice containing MDA-MB-231 cells, effectively inhibited tumor progression.	[74]
		AgNPs-Chi	800 nm (6.34 W.cm^−2^), 36 s	MCF7 and MDA MB-231 cells did not survive, after PTT with prismatic AgNPs-PVP at a concentration of 50 µg.mL^−1^.	[18]
	Melanoma	AgNPs-TiO_2_	808 nm (2 W.cm^−2^), 1 min	Prismatic AgNPs coated with TiO_2_ (200 μg.mL^−1^) presented a η of 60.5% when exposed to 808 nm laser for 6 min. AgNPs-TiO_2_ (100 μg.mL^−1^), under NIR irradiation for 1 min, exhibited high toxicity for B16F10 cells *in vitro*, reducing cell viability to less than 4%, as well as in *in vivo* studies.	[75]
		AgNPs-BSA-ICG-PEG	885 nm (1.3 W), 20 min	AgNPs-BSA-ICG-PEG (30 μmol.L^−1^ as Ag) presented a high temperature rise under NIR irradiation, and showed great anticancer activity against B16F10 cells.	[76]
		AgNPs-BSA	690 nm (1.5 W.cm^−2^), 10 min	The PTT effect of AgNPs-BSA (2.7 mmol.L^−1^ as Ag) was evaluated on B16F10 cells, showing a decrease in viability, with almost complete cell death in temperatures above 45 °C (corresponding to a laser power higher than 0.9 W).	[77]
		AgNPs-BSA-hydrogels	885 nm (1.3 W), 5 min	Hydrogels with AgNPs-BSA (125 μg.mL^−1^ as Ag) exhibited a temperature rise of around 43 °C under 885 nm laser irradiation. These hydrogels showed PTT efficacy, decreasing the viability of B16F10 cells in both *in vitro* and *in vivo* models.	[78]
		AgNPs-PhA	808 nm (1.2 W.cm^−2^), 10 min	The treatment of B16F10 cells with AgNPs-PhA (8 μg-mL^−1^) and NIR light showed a significant reduction in cell viability, which was attributed to simultaneous PTT and PDT. This system was also effective in *in vivo* phototherapy.	[79]
	Cervical Cancer	AgNPs-Dap	808 nm (1.75 W.cm^−2^), 10 min	AgNPs-Dap (1 mL) exhibited a η of 36.8%. The AgNPs-Dap system presented good anticancer toxicity in HeLa cells when exposed to NIR light.	[13]
		AgNPs@Au	808 nm (1.0 W.cm^−2^), 20 min	Prismatic AgNPs coated with Au showed a η = 67% under NIR irradiation for 10 min. *In vivo* studies on mice with HeLa cells-induced tumors showed that AgNPs@Au (100 mmol.L^−1^ in terms of Ag) increased the tumor tissue temperature and induced tumor necrosis.	[80]
		AgNPs-FCO-BSA-FA	808 nm (2.0 W.cm^−2^), 3–5 min	AgNPs-FCO-BSA-FA (80 μg.L^−1^) showed a temperature increase under irradiation with an 808 nm laser. This composite was associated with DOX and exhibited a photo-chemotherapy effect in HeLa cells, killing 90% of the cells.	[81]
		AgNPs-Chi-FA	808 nm (2.0 W.cm^−2^), 6 min	Prismatic AgNPs-Chi-FA (15 μmol.L^−1^) showed a higher temperature increase under NIR irradiation than gold nanorods (50 μmol.L^−1^). The AgNPs-Chi-FA associated with several anticancer drugs under PTT conditions, showed a reduction of 35% in HeLa cells’ viability.	[82]
	Hepatocellular Carcinoma	AgNPs-PyOH	840 nm (1.0 W), 10 min	HepG2 cells’ viability decreased to 20% after PTT treatment with AgNPs-PyOH (0.1 mmol.L^−1^). This system also presented a significant photothermal conversion effect when applied *in vivo*, with almost complete tumor growth inhibition.	[83]
		AgNPs-PDA-GOx	808 nm (1 W.cm^−2^), 3 min	The AgNPs-PDA-GOx (at a PDA concentration of 0.1 mg.mL^−1^) presented a η = 30.2%, when exposed to an 808 nm laser for 5 min. AgNPs-PDA-Gox exhibited a toxic effect against Hepa 1–6 cells.	[84]
	Ovarian Cancer	AgNPs-Z_HER2_	465 nm (95 mW.cm^−2^), 25 min	The spherical AgNPs prepared in the presence of plant extracts (2.2 mg.mL^−1^) showed a temperature increase of around 10 °C. AgNPs conjugated with anti-HER2 affibody were shown to be effective PTT tools against SKOV3-1ip cells.	[85]
	Pancreatic Cancer	AgNPs-IgG	808 nm (2.0 W.cm^−2^), 2 min	AgNPs-IgG (50 μmol.L^−1^), when irradiated, induced the apoptosis of PANC-1 cells and the collapse of the Golgi complex. The viability of these cells decreased to 21.9% post-PTT treatment.	[86]
	Laryngeal Cancer	AgNPs-PVP	490 nm (200 J), 5 h	Spherical AgNPs-PVP (0.3 mmol.L^−1^) were irradiated with a 490 nm laser to treat laryngeal carcinoma cells (Hep-2), decreasing the viability of these cells by 50% after 5 h.	[87]
	Lung Cancer	AgNPs-CuSe	1064 nm (1 W.cm^−2^), 5 min	AgNPs were deposited on the surface of CuSe and this composite (50 μg.mL^−1^) exhibited a η = 52.7% under NIR irradiation for 5 min. The AgNPs-CuSe composites presented cytotoxicity for lung cancer cells under 1064 nm irradiation. *In vivo* studies showed a tumor weight reduction after PTT.	[88]

* irradiation time not indicated. Abbreviations: 5-ALA—5-aminolevulinic acid; AIE—aggregation-induced emission molecule; BSA—bovine serum albumin; CD—carbon dot; Chi—chitosan; Cur—curcumin; CyOH—near-infrared dye; Dap—daptomycin; DOX—doxorubicin; FA—folic acid; FCO—fluorinated carbon fiber oxide; GOx—glucose oxidase; HSA—human serum albumin; ICG—indocyanine green; IgG—immunoglobulin G; PCN—porphyrinic porous coordination network; PDA—polydopamine; PEG—polyethylene glycol; PhA—pheophorbide A; PPA—porcine pancreatic α-amylase; PS—photosensitizer; PVA—polyvinyl alcohol; PVP—polyvinylpyrrolidone; PyOH—pyridine derivative; QRC—quercetin; ROS—reactive oxygen species; UCNPs—upconversion nanoparticles; Z_HER2_—HER2 affibody; ZnPc—zinc phthalocyanine; η—photothermal conversion efficiency.

### 3.2. Photothermal Therapy of Cancer Cells

New therapeutic approaches have been explored using nanomedicine as an alternative to conventional treatments, aiming to reduce associated side effects. Among these emerging strategies, PTT has emerged as a therapeutic technology for cancer treatment, presenting minimal invasiveness, low toxicity, and spatiotemporal selectivity. PTT is based on irradiating photothermal agents (PTAs) with light, particularly in the NIR region [89,90]. The effectiveness of the PTT process depends directly on the PTA used, in which metallic nanoparticles stand out. These NPs convert light into heat through the effect of LSPR, resulting in a local temperature increase and triggering the death of pathogenic cells through hyperthermia. Furthermore, NPs can be functionalized to specifically target cancer cells, thus minimizing damage to healthy tissues [91,92]. AgNPs appear to be the best option compared to other metallic nanoparticles, as they combine good features in terms of the plasmonic effect, which provides more effective light absorption owing to their strong light scattering and surface plasmonic strength, practical synthesis methodologies, high control over size and morphology, and good cost-effectiveness. In addition, they are particularly noteworthy due to their ability to release Ag^+^ ions, which present cytotoxicity and contribute to cellular damage [92,93].

PTT treatment of breast cancer cells using AgNPs has shown promising results, even in triple-negative breast cancer (TNBC), one of the most aggressive and poorly diagnosed breast tumors. Most works employed prismatic AgNPs with an absorption maximum in the NIR, without specific NPs’ surface functionalization [18,72,74]. Nevertheless, some studies have added a targeting molecule, such as folic acid (FA) [71], or a biopolymer, to increase system biocompatibility [68,73]. Furthermore, various studies have associated AgNPs with chemotherapeutic drugs to achieve synergistic therapy with improved outcomes (Table 1) [69,71,73].

Lee et al. [82] used prismatic AgNPs with an absorption maximum of 715 nm, and coated them with chitosan conjugated to folic acid (Chi-FA). FA was selected as the target molecule, to enhance NPs’ accumulation in cells. In this system, an anticancer agent (docetaxel—DTX, paclitaxel—PTX, diallyl disulfide—DADS, or *S*-allylcysteine—SAC) was added. The encapsulation efficiency was higher for PTX (~99%) and lower for SAC (~2%). Viability studies showed that these AgNPs–Chi–FA–drug systems (15 μmol.L^−1^ related to Ag) exhibited toxicity from 40 to 50%, in three tumor types (human gastric adenocarcinoma—AGS, HeLa, and human colorectal adenocarcinoma—HT-29). For comparison, the authors also prepared gold nanorods (AuNRs) with the same Chi-FA coating and anticancer drugs. However, the PTT assays demonstrated that AgNPs-Chi-FA (15 μmol.L^−1^) showed a greater temperature increase compared to AuNRs (50 μmol.L^−1^) when exposed to an 808 nm laser (2 W.cm^−2^) for 6 min. Under PTT conditions, HeLa cell viability was reduced to 47% when incubated with the AgNPs–Chi–FA–drug (1.5 μmol.L^−1^), while without laser irradiation at this NP’s dose, the cell viability remained above 80% [82].

**Figure 6:**
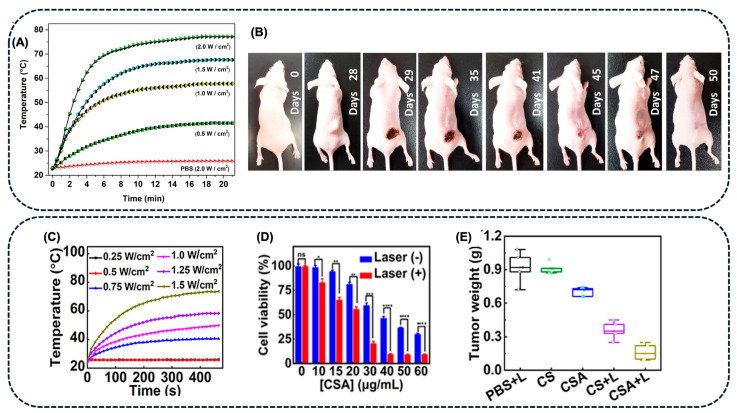
PTT studies with AgNPs-PVA (140 μg.mL^−1^): (**A**) temperature change under irradiation with an 808 nm laser at different power densities; and (**B**) photographs of tumor-bearing mice from tumor inducing (day 0), PTT treatment (day 28), to complete healing (day 50). Reprinted from Mondal et al. [74], Copyright (2022), with permission from Elsevier. Photothermal studies with AgNPs-CuSe (CSA): (**C**) temperature rise when varying the 1064 nm laser power densities; (**D**) lung cancer cells’ viability treated with difference CSA concentrations and without (−) and with (+) laser irradiation, * *p* < 0.05, ** *p* < 0.01, *** *p* < 0.001, **** *p* < 0.0001; and (**E**) tumor weight of mice under different PTT treatments. Reprinted from Ling et al. [88], Copyright (2024), with permission from the American Chemical Society.

A comprehensive computational approach to estimate plasmonic heat generation during PTT guided by photoacoustic imaging (PAI) for cancer treatment was developed by Mondal et al. [74]. Polyvinyl alcohol biopolymer-coated prismatic AgNPs (AgNPs-PVA) were synthesized and studied for PTT and PAI-guided treatment of TNBCs (MDA-MB-231). The prismatic AgNPs-PVA presented a broad absorption band, with a maximum of around 900 nm, and photothermal conversion efficiency (η) of 30.4%, using an NP dose of 140 μg.mL^−1^ and 7 min of irradiation with an 808 nm laser at an irradiance of 1.0 W.cm^−2^ (Figure 6A). The data from the computational simulations were in agreement with the experimental results, indicating that these theoretical models could be used to predict photothermal heat generation. AgNPs-PVA exhibited excellent biosafety without irradiation with NIR light and strong efficiency as a PAI contrast agent. However, under NIR irradiation (1.0 W.cm^−2^) for 7 min, AgNPs-PVA (140 μg.mL^−1^) were toxic to TNBC, reducing cell viability to 5%. In *in vivo* studies, animals treated with AgNPs-PVA (140 μg.mL^−1^) and exposed to NIR irradiation (1.0 W.cm^−2^, 7 min) presented effective tumor inhibition without further recurrence (Figure 6B) [74].

In another work, Liu et al. [18] used prismatic AgNPs coated with chitosan (Chi) to kill breast cancer cells (MCF-7 and MDA MB-231 cells), both non-infected and infected with *Pseudomonas aeruginosa*. AgNPs-Chi exhibited a strong absorption at 800 nm, an average hydrodynamic diameter of 79 nm, and a Zeta potential of +18 mV. Under exposure to an 800 nm (5 W) laser irradiation, AgNPs-Chi increased the local temperature, with a minimum concentration of 10 μg.mL^−1^ required to exceed 45 °C, sufficient for thermal ablation of tumor cells. *In vitro* studies showed that the viability of breast cancer cells (non-infected and infected with bacteria) was reduced following incubation with AgNPs-Chi. Photothermal treatment of these cells was carried out by incubating them with AgNPs-Chi for 2 h and 36 s of NIR irradiation. After 24 h of PTT with a AgNPs dose of 10 μg.mL^−1^, non-infected breast cancer cells presented a considerable viability reduction, while infected cells retained over 32% viability. However, when the AgNP dosage was increased to 25 μg.mL^−1^, less than 10% of all the cells (infected and non-infected) survived [18].

Combined chemo- and photothermal therapy is a strategy of increasing interest, which could overcome the limitations of each therapy and improve treatment outcomes. In this context, AgNPs have been used as PTAs and/or drug delivery platforms. A common drug used in chemotherapy is doxorubicin (DOX), which was associated with AgNPs by Carrese et al. [69], to target breast cancer (HS578T cells). For this, DOX was loaded into AgNPs coated with human serum albumin (HSA), to obtain the therapeutic agent AgNPs-HSA-DOX. This system (1 μg.μL^−1^) presented a photothermal efficiency of 30% when exposed to 808 nm laser irradiation for 15 min. Confocal microscopy confirmed the uptake of AgNPs-HSA-DOX by HS578T cells, which also presented greater cytotoxicity compared to free DOX. PTT treatment (808 nm, 3 W.cm^−2^, 5 min) enhanced even more the cytotoxic activity of AgNPs-HSA-DOX against HS578T breast cells compared to pure chemotherapy treatment [69].

Some authors have proposed therapeutic approaches for skin cancer based on AgNPs coated with bovine serum albumin (BSA). Kim et al. [77] prepared AgNPs-BSA and evaluated its anticancer activity in B16F10 melanoma cells, under 690 nm laser irradiation (1 W for 10 min). AgNPs-BSA were obtained with an absorption maximum of 420 nm, a diameter of around 100 nm, and a spherical shape. AgNPs-BSA were incubated with B16F10 cells and showed cytotoxicity, with an IC50 of 66 ± 9 μmol.L^−1^, without light irradiation, indicating the production of ROS and inducing oxidative stress in cells. When irradiated with a 690 nm laser (0.9 W for 10 min), the AgNPs-BSA (3.6 mmol.L^−1^) afforded almost complete cell mortality, achieving a temperature of 45 °C. In a following study, the same research group [78] incorporated AgNPs-BSA in gelatin and glycerin to produce hydrogels, and evaluated their cytotoxicity in B16F10 cells under irradiation with an 885 nm laser (1.1 W) for 5 min. The hydrogel films (with 200 μg.cm^−2^ of Ag content) presented an IC50 of 96.1 μmol.L^−1^ (without irradiation), and achieved the photothermal temperature (45 °C) with a laser power of 0.5 W, reducing to 8% cell viability. AgNPs-BSA hydrogels were also analyzed *in vivo* and topically applied to B16F10 tumor-bearing mice. The authors found that hydrogels applied to tumor skin regions increased the local temperature under PTT conditions (885 nm, 5 min). However, it required a laser power of 0.6 W to observe a significant tumor reduction [77].

A synergistic approach associating PTT and PDT was explored by Patil et al. [79] for melanoma treatment. In this work, AgNPs were produced in the presence of pheophorbide A (PhA), yielding AgNPs-PhA. These spherical NPs presented a broad absorption band with a maximum at 850 nm and an average size of around 40–50 nm. Photothermal conversion studies under 808 nm laser irradiation (1.2 W.cm^−2^) for 10 min, showed that bare AgNPs exhibited a greater temperature increase than AgNPs-PhA, with the response dependent on both nanoparticle dosage and laser intensity. These authors also verified that, when exposed to NIR, AgNPs-PhA release PhA, followed by its degradation. *In vitro* assays using B16F10 melanoma cells showed cytotoxicities of 68 and 13% for AgNPs and AgNPs-PhA (8 µg.mL^−1^), respectively, under NIR irradiation. Additionally, AgNPs-PhA presented minimal cell damage to L929 cells. Further studies demonstrated the intracellular ROS spawning mediated by NIR irradiation, which was attributed to the presence of PhA, confirming the synergistic effect between PTT and PDT. The antitumoral efficiencies of these AgNPs and AgNPs-PhA were also evaluated *in vivo*, using B16F10 tumor-bearing mice under NIR irradiation. However, AgNPs showed a higher temperature rise at the tumor site, and AgNPs-PhA presented greater tumor inhibition potential, due to the combination of hyperthermia and ROS generation [79].

For hepatocellular carcinoma treatment, Yu et al. [84] proposed a platform containing AgNPs, polydopamine (PDA), and glucose oxidase (GOx), with enhanced photothermal conversion efficiency. GOx was added to improve the cell sensitivity to PTT by catalyzing the glucose present, since this enzyme alters the cell’s metabolism and reduces the production of cellular heat shock proteins. Moreover, the authors observed that the catalytic reaction of glucose by GOx produced ROS, which led to the release of Ag^+^, thereby enhancing cellular toxicity. The AgNPs-PDA-GOx (0.1 mg.mL^−1^ relative to PDA) was evaluated for PTT efficacy using an 808 nm laser (1 W.cm^−2^) for 5 min, achieving a photothermal conversion efficiency of 30.2%. Then, the authors conducted *in vitro* experiments by incubating mouse hepatoma cells (Hepa 1-6) with AgNPs-PDA-GOx for 4 h, followed by 3 min of laser irradiation. The results demonstrated that the presence of AgNPs-PDA-GOx (50 μg.mL^−1^ of PDA and 1.5 mg.L^−1^ of GOx) significantly enhanced the cytotoxic effect, killing more than 80% of the cells. The same behavior was observed in *in vivo* experiments with mice, where the injection of AgNPs-PDA-GOx increased the tumor’s local temperature and led to tumor thermal ablation [84].

To treat lung cancer, Ling et al. [88] prepared CuSe nanocubes covered with AgNPs for enhanced synergistic cancer therapy by PTT and ROS spawning. The authors observed that, under 1064 nm laser irradiation for 5 min, the AgNPs-CuSe (45 μg.mL^−1^) presented a good photothermal conversion with a η = 52.7% (Figure 6C). Moreover, in the presence of H_2_O_2_ and exposure to NIR irradiation, this composite showed enhanced ROS generation. The cytotoxicity of CuSe and AgNPs-CuSe was evaluated *in vitro* with lung cancer cells (A549 cells), with and without irradiation with NIR light. The results showed a dose-dependent cell apoptosis that was intensified by the 1064 nm laser irradiation (Figure 6D). *In vivo* assays were carried out using A549 tumor-bearing mice models, the AgNPs-CuSe composites (5 mg.Kg^−1^) were injected intratumorally, followed by the irradiation with NIR light (1064 nm, 5 min). The results showed a temperature increase to 54 °C at the tumor site and a reduction in the tumor weight, 14 days after the treatment (Figure 6E) [88].

### 3.3. Phototherapy for the Inactivation of Bacteria

Photoinactivation of microorganisms has been made by PDT or PTT strategies. The use of PDT for inactivating microorganisms has been called photodynamic inactivation (PDI) or antimicrobial photodynamic therapy (aPDT), though it follows the same principles as PDT. A PS is irradiated by an adequate light source, which will yield ROS in the presence of molecular oxygen, leading to cellular death. This aPDT approach has shown promising results in overcoming the microorganism resistance to conventional treatments [94,95].

In recent years, there has been an increase in reports of bacteria with mechanisms of antibiotic resistance, which consequently hinder the treatment of infectious processes. These protective mechanisms are diverse and may include efflux pumps that expel drugs, enzymes that degrade antibiotics, alterations in metabolic pathways, and the formation of biofilms [96]. The rise in infections caused by bacteria resistant to conventional treatments poses a significant threat to global health and necessitates increasingly specialized therapeutic approaches. In this context, research is being conducted into alternatives that either replace or complement traditional antibiotics such as the use of AgNPs [94,95]. 

AgNPs have been extensively studied as antimicrobial agents for many applications, like treating infections, wound healing, and surgical tools. Recently, the evaluation of the synergistic effect of AgNPs and light has been increasing in the literature. Several studies have demonstrated that AgNPs’ antibacterial effect can be enhanced by their association with light irradiation, inhibiting or suppressing bacterial growth. Several research studies have also focused on developing methodologies to inhibit the growth of biofilms formed by bacteria, which are microbial communities that adhere to surfaces encased in a self-produced extracellular polymeric matrix. This matrix provides significant protection to microorganisms, shielding them from external stressors and hindering the penetration of antibiotics, thus complicating treatment strategies [97].

Phototherapy approaches (aPDT and PTT) based on AgNPs have been applied for Gram-positive and Gram-negative bacteria, mainly in *Staphylococcus aureus*, *Escherichia coli*, *Pseudomonas aeruginosa*, and *Klebsiella pneumoniae*. Additionally, some works linked AgNPs with PSs, like Pps, methylene blue (MB), and phthalocyanines, to explore the aPDT approach. A summary of these works that use AgNPs and light to inactivate bacteria can be found in Table 2.

A common PS used in aPDT to inactivate bacteria is porphyrin derivatives, which have great photosensitivity. Morales de-Echegaray et al. [98] associated AgNPs with gallium-substituted hemoglobin (AgNP-GaHb) by using a non-covalent strategy, to inactivate *Staphylococcus aureus* and methicillin-resistant *S. aureus* (MRSA). The produced AgNPs-GaHb presented an absorption maximum at 396 nm and a spherical shape with a mean diameter of 17 nm. The aPDT studies revealed that bacterial strains were eradicated after 10 s of irradiation with a 405 nm LED light source, with a AgNPs-GaHb dose of 5.8 and 16.3 μg.mL^−1^ for *S. aureus* and MRSA, respectively. These authors also demonstrated the superior PDI effect of the AgNPs-GaHb system when compared with the individual components [98].

Similarly, Openda et al. [99] investigated the synergistic effect of AgNPs and Pps in aPDT. The porphyrin derivative was coordinated with three cations (Zn^2+^, Ga^2+^, and In^3+^), then the Pps were covalently conjugated with carbonaceous nanodiamonds (NDs), followed by the addition of AgNPs. aPDT assays were performed on *S. aureus* using irradiation at 415 nm for 2 h and a dose of 10 μg.mL^−1^ of AgNPs-ND-Pp. These results showed that a complete bacterial inactivation was observed within 120 min for the zinc PS, while for the Ga and In conjugates, the *S. aureus* eradication was observed after 30 min, achieving a reduction of up to 10.48 ± 0.003 log_10_. However, only In and Ga-containing Pps (100 μg.mL^−1^) were able to photo-inactivate *S. aureus* biofilms. The authors suggested that the functionalization of AgNPs with NDs and porphyrins facilitates drug binding to the bacterial cell wall, enhancing intracellular ^1^O_2_ production and promoting bacterial cell death [99]. 

Methylene blue, for instance, has also demonstrated potential in aPDT when combined with AgNPs. Rodrigues et al. [39] investigated the AgNPs-MB conjugate applied to *S. aureus* strains isolated from mastitis cases. In this work, the AgNPs were prepared with a prismatic shape to tune their plasmonic activity to the MB absorption region (600–700 nm), and the AgNPs-MB were linked through electrostatic interactions. Then, using a 660 nm laser, the AgNPs-MB were irradiated for up to 300 s, with intervals of either 20 or 60 s. The results revealed that continuous irradiation for 60 s generated significant ROS levels (89.7 ± 0.6%). When performing aPDT assays on *S. aureus* strains, the authors observed a bacterial reduction of 2 log_10_ after 3 min of irradiation, reaching complete inactivation after 6 min [39].

**Figure 7:**
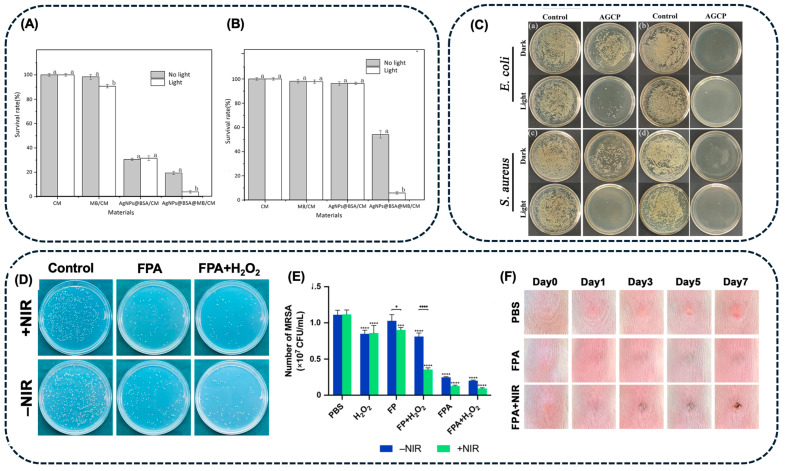
Antibacterial activity of chitosan microspheres (CS) containing AgNPs-BSA-MB against (**A**) *E. coli* and (**B**) *S. aureus* strains, under a 660 nm light irradiation for 20 min. ^a,b^ *p* < 0.05. Reprinted from Ren et al. [100], Copyright (2023), with permission from the American Chemical Society. (**C**) Antibacterial effect of AgNPs-Cur (AGCP) under blue light irradiation in *E. coli* and *S. aureus* bacteria. Reprinted from Liu et al. [101], Copyright (2024), with permission from Elsevier. AgNPs-Fe_2_O_3_-PDA (FPA) effect on MRSA bacteria in the absence (−) or presence (+) of NIR irradiation (**D**,**E**) *in vitro* and (**F**) *in vivo*. * *p* < 0.05, *** *p* < 0.001, **** *p* < 0.0001. Reprinted from Hong et al. [102], Copyright (2024), with permission from Springer Nature.

The use of AgNPs-MB in aPDT was also explored by Ren et al. [100]. These authors synthesized AgNPs-BSA, encapsulated them into chitosan microspheres (CS), and subsequently incorporated MB into the AgNPs-BSA-CS via adsorption. Their study demonstrated that AgNPs-BSA-CS-MB presented an enhancement of 87 and 94% in inactivating *E. coli* and *S. aureus* strains, respectively, compared to AgNPs alone, with a 660 nm light source for 20 min (Figure 7A,B). The synergistic effect of AgNPs and MB in aPDT was attributed to the ability of MB to form three distinct species (MB^+^, MB, and MBH) and its conjugated structure, which facilitates charge transfer and electron delocalization. Upon light exposure, MB absorbs energy and transitions to an excited state, generating free radicals that convert molecular oxygen into singlet oxygen. These radicals, possessing strong oxidative properties, promote the conversion of AgNPs into Ag^+^ ions, which are released into the solution [100]. 

Curcumin (Cur), a photoactive polyphenolic compound, is also a common PS for aPDT, taking advantage of being a naturally occurring molecule. Liu et al. [101] investigated the combination of AgNPs with Cur to inactivate *E. coli* and *S. aureus* bacteria (Figure 7C). The results showed that the presence of AgNPs-Cur markedly enhances ROS yield under blue light irradiation for 10 min. When these bacteria were treated with Cur under blue light irradiation, inhibition rates of 51.5% for *E. coli* and 63.7% for *S. aureus* were observed. However, the AgNPs-Cur conjugates demonstrated significantly higher antibacterial activity under the same conditions, with inhibition rates of 97.9% for *E. coli* and 99.5% for *S. aureus*. This enhanced activity was attributed to the rapid generation of ROS mediated by Cur. Prolonged antibacterial efficacy was observed after 12 h of treatment, with the gradual release of Ag^+^ potentiating the bactericidal effect, ultimately achieving complete bacterial inhibition. This outcome was attributed to the synergistic action of the initial ROS production and the sustained release of Ag^+^, which caused extensive damage to bacterial structures, including membranes and cell walls, leading to bacterial death [101].

PTT approaches using AgNPs for inactivating bacteria have been less used compared to aPDT, and this methodology has been growing. The literature highlights numerous advantages of PTT in pathogen treatment, including its broad-spectrum antibacterial activity, the ability to penetrate tissues without causing significant damage (for NIR irradiation), localized application through precise temperature increases, and a reduced risk of inducing bacterial resistance [103].

Pistonesi et al. [104] investigated the photothermal activity of titanium alloys functionalized with prismatic AgNPs (AgNPs-TiO_2_). Using a 904 nm laser to induce hyperthermia, the study demonstrated that this system (14 µg.mL^−1^) presented a photothermal conversion efficacy (η) of 35%, and effectively reduced *S. aureus* viability and adhesion. However, the specific contribution of the prismatic AgNPs did not lead to significant enhancements in the overall performance, indicating that the alloy’s base material played a more prominent role in achieving these results [104].

Another significant advance in the use of PTT to combat resistant bacterial infections was demonstrated by Mechouche et al. [105], who synthesized AgNPs via a biosynthetic method using the *Streptomyces tuirus* S16 strain. Optimization of the synthetic conditions was carried out by evaluating the concentration of various reagents in the culture media, as well as reaction time and temperature. The resulting AgNPs exhibited an absorption maximum at 420 nm, an average diameter of 23 nm, and a zeta potential of −19.7 mV. Antibacterial assays against *E. coli* demonstrated the nanoparticles’ effectiveness both with and without visible light exposure. Treatment with AgNPs at 105 μg.mL^−1^ in the absence of light resulted in a 60% reduction in bacteria optical density, whereas exposure to a lower dose (52.5 μg.mL^−1^) under visible light irradiation (0.2 W.cm^−2^ for 2 h) led to an approximately 80% reduction, indicating enhanced bactericidal activity under light stimulation [105].

Several researchers reported a synergistic therapeutic strategy for bacterial infections, combining PTT and chemodynamic therapy (CDT). Zhou et al. [106] synthesized a flower-shaped nanocomposite composed of AgNPs, phosphotungstic acid (POM) for CDT, and PDA for PTT. AgNPs-PDA-POM (200 μg.mL^−1^) presented a η of 38% when exposed to an 808 nm laser (0.75 W.cm^− 2^) for 6 min. This system also presented a peroxidase-like activity, catalyzing H_2_O_2_, confirming its potential for CDT. For *in vivo* applications, the AgNPs-PDA-POM nanoflowers were incorporated within a Chi and gelatin hydrogel, to obtain multifunctional wound dressing scaffolds. AgNPs-PDA-POM (700 μg.mL^−1^) scaffolds were evaluated against Gram-negative *E. coli* and Gram-positive *S. aureus*, under NIR irradiation for 10 min. *In vitro* assays showed that only NIR irradiation or the addition of H_2_O_2_ was not effective in killing the bacteria. However, a complete inactivation of both bacteria was achieved by combining NIR light and H_2_O_2_, indicating a synergy between PTT and CDT. The *in vivo* application of the AgNPs-PDA-POM scaffolds showed that this nanocomposite promoted wound healing by inactivating the bacterial infection, combining PTT and CDT [106].

A study by Hong et al. [102] proposed the association of AgNPs with Fe_2_O_3_ NPs coated with PDA, to achieve a synergistic PTT and CDT antibacterial treatment, to eradicate MRSA. The AgNPs-Fe_2_O_3_-PDA core–shell structure presented a hydrodynamic size of around 335 nm, containing spherical AgNPs with 50 nm (determined by TEM). The AgNPs-Fe_2_O_3_-PDA (1 mg.mL^−1^, 808 nm, 1 W.cm^−2^, 500 s) presented a η = 30.1%; however, AgNPs exhibited a shielding effect, decreasing the temperature rise of AgNPs-Fe_2_O_3_-PDA when compared with Fe_2_O_3_-PDA. Nevertheless, AgNPs-Fe_2_O_3_-PDA presented a higher ROS yield in the presence of H_2_O_2_, which is crucial for CDT. The PTT-CDT effect of these NPs was evaluated *in vitro*, indicating a complete bacteria inactivation post-treatment with AgNPs-Fe_2_O_3_-PDA (200 µg.mL^−1^), H_2_O_2_, and 808 nm NIR laser irradiation (1 W.cm^−2^, for 10 min) (Figure 7D,E). This system was also efficient in controlling bacterial infections caused by MRSA, in animal models (Figure 7F) [102].

Some authors have studied the synergistic combination of aPDT and PTT to inactivate *S. aureus* and *E. coli* bacteria strains. For instance, Bourgonje et al. [107] observed that prismatic AgNPs–citrate (λ_abs_ = 800 nm, 5 mg.L^−1^), when irradiated with NIR light (810 nm, 0.99 W.cm^−2^) for 15 min, exhibited an excellent antibacterial activity. By investigating the antibacterial mechanism, the authors detected that ROS generation, the release of Ag^+^, and plasmonic heating were involved. These authors also observed that by irradiating this system with a 405 nm laser, the bacteria’s eradication was lower [107].

In another study, Liu et al. [108] associated AgNPs with Cur, a known PS for PDT, and evaluated the synergistic aPDT and PTT effect of this system in *S. aureus* and *E. coli.* To improve the system stability, AgNPs-Cur were loaded into montmorillonite (Mt), a material known for its biocompatibility and high surface area, and then AgNPs-Cur-Mt were encapsulated into a hydrogel. The irradiation of AgNPs-Cur-Mt with 405 nm laser (0.4 W.cm^−2^) indicated the spawning of ROS, which was attributed to the presence of Cur. On the other hand, exposing AgNPs-Cur-Mt to 808 nm light (0.9 W.cm^−2^) resulted in a temperature rise due to the presence of AgNPs. Then, the antibacterial activity of AgNPs-Cur-Mt was studied with *S. aureus* and *E. coli*, under irradiation. It was observed that *E. coli* was less sensitive to aPDT than *S. aureus*. Nevertheless, a toxicity higher than 99% was achieved for the two bacteria by simultaneous irradiation with 405 (0.4 W.cm^−2^) and 808 nm lasers (1.2 W.cm^−2^), for 20 and 8 min, respectively. This system was also successful in the elimination of *S. aureus* biofilm [108].

**Table 2:** Summary of recent works combining AgNPs and light irradiation for bacteria inactivation and wound healing, from the last 5 years.

Bacteria	Therapy Approach	System	Phototherapy Details	Results	Refs.
*Staphylococcus aureus*	aPDT	AgNPs-GaHb	405 nm (140 mW.cm^−2^), 10 s	Eradication of *S. aureus* and MRSA was achieved using, respectively, 5.8 and 16.3 μg.mL^−1^ of AgNPs-GaHb and exposure to 405 nm laser for 10 s.	[98]
		AgNPs–PpIX–polymer	635 nm, 10 min	*S. aureus* bacteria were inactivated both *in vitro* and *in vivo* using, respectively, 400 μg.mL^−1^ and 1 mg.mL^−1^ of AgNPs–PpIX–polymer.	[109]
		AgNPs-ND-Pp	415 nm (15.6 μW.mm^−2^), 30 min	Ga and In-based Pps conjugated with AgNPs showed bacteria inactivation with a dose of 10 μg.mL^−1^ and a biofilm reduction with 100 μg.mL^−1^, under 30 min of light exposure.	[99]
		AgNPs-MB	660 nm (45.8 mW∙cm^−2^), 6 min	Prismatic AgNPs were associated with MB, and aPDT assays showed a complete inactivation of *S. aureus* after 6 min of irradiation.	[39]
		AgNPs-ND-Pc	670 nm (524 mW.cm^−2^), 2 h	The conjugates improved the aPDT effect, showing a log reduction of 5.1–5.3 in biofilms.	[110]
		AgNPs-MOF	Visible light (100 mW.cm^−2^), 1 h	AgNPs-MOF showed a satisfactory aPDT effect, presenting the ability to inactivate bacteria in both *in vitro* and *in vivo* models.	[111]
	PTT	AgNPs-TiO_2_	904 nm (83.3 mW.cm^−2^), 10 min	Prismatic AgNPs associated with TiO_2_ achieved a η = 35%. AgNPs-TiO_2_ (14 μg.mL^−1^) were able to inhibit colony formation, under irradiation.	[104]
		AgNPs-PDA-Fe_3_O_4_	808 nm (1.0 W.cm^− 2^), 10 min	The system presented a η = 30% and, under NIR irradiation, AgNPs-PDA-Fe_3_O_4_ presented a great bactericidal activity, causing damage to the bacterial cell wall.	[102]
*Pseudomonas aeruginosa* and *S. aureus*	aPDT	AgNPs-MB	660 nm (467 mW.cm^−2^), 3 min	AgNPs-MB (125 μg.mL^−1^) exhibited a 4.3-log10 and 1.1-log10 CFU.mL^−1^ reduction on *P. aeruginosa* and *S. aureus* after aPDT assays.	[112]
MSRA and *Klebsiella pneumoniae*	aPDT	AgNPs and TMPyP	414 nm (54 mW.cm^−2^), 5 h	The MIC was reduced for aPDT in the presence of AgNPs and TMPyP.	[113]
*Bacillus subtilis*	PTT	AgNPs-PVP	795 nm (1.35 W.cm^−2^), 5 min	Prismatic AgNPs-PVP (3.5 μg Ag.mL^−1^) achieved a complete eradication of bacteria (5 × 10^7^ CFU.mL^−1^) under laser irradiation for 5 min.	[114]
*Escherichia coli*	PTT	AgNPs	Visible light (0.2 W.cm^−2^), 30 min	Biosynthesized AgNPs (52.5 μg.mL^−1^) under visible light irradiation were able to completely inactivate *E. coli* strains after 30 min.	[105]
*Staphylococcus aureus* and *Escherichia coli*	aPDT	ZIF8-PAA-MB-AgNPs-Van-PEG	650 nm (202 mW), 5 min	*In vitro* and *in vivo* antibacterial assays showed the efficiency of the AgNPs-MB system in inactivating *E. coli*, *S. aureus*, and MRSA by aPDT.	[115]
		AgNPs-MB	660 nm, 5 min	AgNPs-MB showed higher aPDT performance than bare MB or AgNPs, resulting in the inactivation of approximately 10^6^ CFU mL^−1^ of *S. aureus* and *E. coli* bacteria.	[116]
		AgNPs-MB	Xenon lamp, 20 min	aPDT studies showed that AgNPs-MB conjugates increased the inactivation of *E. coli* and *S. aureus* strains by 87% and 94%, respectively, compared to bare AgNPs.	[100]
		AgNPs-Ce6	660 nm (20 mW.cm^−2^), 20 min	AgNPs-Ce6 (1 mg.mL^−1^) under PDT conditions can completely eradicate *S. aureus* and *E. coli* biofilms.	[117]
		AgNPs–extract	405 nm (20 mW.cm^−2^), 3 min	Treatment of bacteria with AgNPs–extract (2 mmol.L^−1^), under red light irradiation for 3 min, showed a viability decrease of 79.5% and 85.0% for *E. coli* and *S. aureus* strains, respectively.	[118]
		AgNPs-GA-Cur-POTS	Blue light, 10 min	This system, where Cur has the PS, showed an antibacterial rate higher than 97% against the two bacteria strains, after light irradiation.	[101]
	PTT	AgNPs–PDA–hydrogel	808 nm (1.0 W.cm^−2^), 5 min	The system showed the ability to inhibit bacterial growth under NIR, but a better result was achieved when H_2_O_2_ was also used.	[119]
		AgNPs-PDA-ZIF8-ICG	808 nm (1.5 W.cm^−2^), 20 min	The composites (100 μg.mL^−1^), upon irradiation, damaged the bacteria membrane due to the hyperthermia and release of Ag and Zn ions.	[120]
		AgNPs–PDA–POM– hydrogel	808 nm (0.75 W.cm^−2^), 6 min	AgNPs-PDA-POM nanoflowers (200 μg.mL^−1^) presented a η = 35% and the same system at 700 μg.mL^−1^ eradicated 90% of both bacteria, under NIR irradiation. However, the hydrogel scaffold only inactivated the bacteria under NIR irradiation and with H_2_O_2_.	[106]
		AgNPs-PDA-Alg-ABA	808 nm (1.0 W.cm^−2^), 5 min	The AgNPs-PDA-Alg-ABA hydrogel exhibited a η of around 49%, under 808 nm irradiation for 5 min. Under laser irradiation, this system inactivated *S. aureus* and *E. coli*, changing their morphology. *In vivo* assays confirmed the good antibacterial performance of this hydrogel.	[121]
		AgNPs–TA–hydrogel	808 nm (133 mW.cm^−2^), 15 min	Hydrogels containing AgNPs-TA were prepared to treat wound infections. AgNPs–TA–hydrogels (with 200 mmol.L^−1^ of Ag) presented enhanced antibacterial and healing properties, when irradiated with NIR.	[122]
		AgNPs–TA–hydrogel	LED 630–850 nm (0.92 W.cm^−2^), 5 min	AgNPs stabilized with tannic acid (TA) were incorporated into a hydrogel for wound healing applications. AgNPs–TA–hydrogels promoted the temperature rising and antibacterial effect when irradiated.	[123]
		AgNPs-Chi-PB	808 nm (1.0 W.cm^−2^), 10 min	AgNPs-Chi-PB, prepared for wound healing, showed a η = 43%. *In vitro* experiments demonstrated that the combination of AgNPs-Chi-PB + NIR irradiation had a stronger effect on both multidrug-resistant pathogens. However, MRSA required a higher dosage (10 μg.mL^−1^) than *E. coli* (3 μg.mL^−1^).	[124]
		AgNPs–lignin	808 nm (1.8 W.cm^−2^), 5 min	AgNPs loaded in lignin composites (1 mg.mL^−1^) presented a η = 29%, under NIR irradiation for 10 min. AgNPs–lignin exhibited antibacterial performance, under NIR irradiation for 5 min, achieving around a 6 log_10_ CFU.mL^−1^ reduction in *E. coli* and *S. aureus*.	[125]
		AgNPs–MT–hydrogel	808 nm (2.0 W.cm^−2^), 5 min	AgNPs prepared with *Mentha pulegium* (MP) extract were incorporated into gelatin hydrogels to treat infected wounds. These AgNPs–MT–hydrogels showed good antibacterial activity and enhanced the wound healing rate, under NIR irradiation, in *in vivo* models.	[126]
	aPDT + PTT	AgNPs–citrate	810 nm (0.99 W.cm^−2^), 15 min	Prismatic AgNPs showed photoinduced antibacterial activity, which was attributed to the ROS yield and plasmonic heating.	[107]
		AgNPs-Cur-Mt	405 (0.4 W.cm^−2^) for 20 min, and 808 nm (1.2 W.cm^−2^) for 8 min	Prismatic AgNPs and Cur were associated to achieve a synergistic system for aPDT and PTT, presenting an antibacterial rate higher than 99% for both bacteria.	[108]

Abbreviations: ABA—3-aminophenylboronic acid; Alg—sodium alginate; Ce6—chlorin e6; Chi—chitosan; Cur—curcumin; GA—gallic acid; GaHb—gallium-substituted hemoglobin; ICG—indocyanine; MB—methylene blue; MIC—minimum inhibitory concentration; MP—*Mentha pulegium* extract; MOF—metal-organic-framework; MRSA—methicillin-resistant *S. aureus*; Mt—montmorillonite; ND—nanodiamond; PAA—polyacrylic acid; PB—Prussian blue; Pc—phthalocyanine; PDA—polydopamine; PEG—polyethylene glycol; POM—phosphotungstic acid; POTS—perfluorosilane; Pp—porphyrin; PpIX—protoporphyrin IX; TA—tannic acid; TMPyP—5,10,15,20-tetrakis(*N*-methylpyriminium-4-yl) porphyrin; Van—vancomycin; ZIF8—zeolitic imidazolate framework; η—photothermal conversion efficiency.

### 3.4. Wound Healing

Wounds are tissue damages that can occur due to various factors, like surgery, trauma, and other disease conditions. The wound healing process involves three well-defined phases: inflammation, proliferation, and tissue remodeling. One of the major concerns in wound management is infection, as the skin hosts a diverse microbial population, and the production of free radicals that cause oxidative stress in the wound site. This concern is especially critical in cases involving diabetic patients or infections by multidrug-resistant bacteria. Thus, AgNPs have been used in wound dressings to overcome this issue, mainly due to their antibacterial activity [127,128]. Recently, AgNPs have been incorporated into hydrogel-based dressings and combined with light irradiation to enhance wound healing rates and prevent bacterial infection (Table 2).

**Figure 8:**
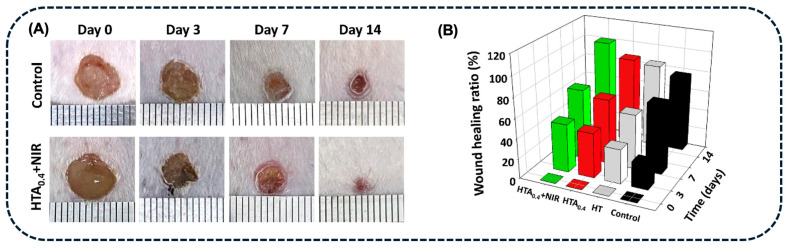
Wound healing properties of AgNPs-TA hydrogels under NIR irradiation (HTA_0.4_ + NIR) *in vivo*: (**A**) mice photographs and (**B**) healing ratio over time following treatment. Reprinted from Chang et al. [122], Copyright (2023), with permission from Elsevier.

Chang et al. [122] developed a hydrogel containing AgNPs for the photothermal treatment of infected wounds. In this study, AgNPs stabilized with tannic acid (TA) were incorporated into a hydrogel composed of hyaluronic acid modified with tyramine. AgNPs-TA were achieved with a spherical morphology, an average size of 9.8 nm, and an absorption band at 420 nm. The photothermal effect of AgNPs–TA–hydrogel was evaluated under 880 nm laser irradiation (0.92 W.cm^−2^ for 5 min), showing a temperature increase that correlated with the AgNPs’ concentration in the hydrogels. Although both blank hydrogels and bare AgNPs-TA presented antibacterial activity against *E. coli* and *S. aureus*, complete eradication of these microorganisms was only achieved with AgNPs–TA–hydrogels (0.4 mg.mL^−1^ of AgNPs-TA) under NIR irradiation. Furthermore, the AgNPs–TA–hydrogels showed enhanced antioxidant activity, improved coagulation capacity, and good biocompatibility. The wound healing efficacy of AgNPs–TA–hydrogels was tested *in vivo* using mice co-infected with *E. coli* and *S. aureus*. The combination of AgNPs–TA–hydrogel and NIR irradiation significantly accelerated the healing process, achieving a 99% wound closure rate within 14 days of treatment (Figure 8A,B) [122].

Shen et al. [124] developed nanocomposites using Prussian blue (PB) coated with chitosan (Chi) as a nanocarrier for anchoring AgNPs, forming the AgNPs-Chi-PB system. When combined with 808 nm NIR laser irradiation, these nanocomposites showed superior efficacy in eliminating *E. coli* and MRSA in *in vitro* studies. AgNPs-Chi-PB showed a minimum inhibitory concentration (MIC) of 6 μg.mL^−1^ against MRSA and 2 μg.mL^−1^ against *E. coli*. Furthermore, by varying the material concentrations (50–200 μg.mL^−1^) and laser power (0.5–1.5 W.cm^−2^), the authors observed that the localized temperature increase could be approximately 20 °C. These authors have also performed studies in diabetic mouse models, observing that AgNPs-Chi-PB + NIR were able to reduce the infection and promote abscess healing [124].

In another approach, Wei et al. [126] synthesized AgNPs using *Mentha pulegium* (MP) extract, and incorporated them into a gelatin hydrogel. The AgNPs-MP were synthesized under UV irradiation, affording spherical nanostructures with an absorption maximum at 410 nm. These AgNPs showed potential for PTT, exhibiting a significant temperature increase upon exposure to an 808 nm laser (2.0 W.cm^−2^). Moreover, AgNPs-MP displayed antibacterial activity, with a minimum inhibitory concentration (MIC) of 200, 200, and 150.0 μg.mL^−1^ against *E. coli*, *S. aureus*, and MRSA, respectively. When the treatment was associated with NIR irradiation for 5 min, the MICs were reduced to 100, 150, and 100 μg.mL^−1^, against *E. coli*, *S. aureus*, and MRSA, respectively, highlighting a synergistic photothermal antibacterial effect. The AgNPs–MP–gelatin hydrogel was used in the treatment of MRSA-infected cutaneous wounds in rats. The group treated with a AgNPs–MP–gelatin hydrogel and NIR showed complete wound healing within 10 days of PTT. In addition, these hydrogels were tested with human umbilical vein endothelial cells (HUVECs), presenting good biocompatibility [126].

### 3.5. Photodynamic Inactivation of Fungi

AgNPs have also been used to innovate fungus infection treatments, using the aPDT approach. For this, AgNPs have been associated with photosensitizers, like porphyrin and phenothiazinium derivatives, and irradiated using visible light. Several therapeutic doses and irradiation times were evaluated. However, the works reported in the literature have *Candida albicans* as their principal target [129,130,131].

Caruso et al. [129] associated AgNPs with phenothiaziniums: MB, new methylene blue N (NMBN), and new methylene blue N with zinc (NMBN-Zn). AgNPs presented an absorption band at 420 nm and a spherical morphology, while the conjugates also showed an absorption maximum between 624 and 655 nm, corresponding to the photosensitizer. The antifungal activity studies, against *C. albicans* and *Fusarium keratoplasticum*, were carried out using LEDs with an emission spectrum between 600 and 650 nm for 16.4 min (15 J.cm^−2^). All conjugates, in the presence of the red light, exhibited a significant reduction in fungi survival. For *C. albicans*, the most efficient system was AgNPs-NMBN-Zn, while AgNPs-NMBN were the best for *F. keratoplasticum* [129].

In another work, Rodrigues et al. [131] associated AgNPs with MB to irradicate *C. albicans*. These authors used prismatic AgNPs with an absorption maximum of around 664 nm, which was well overlapped with the MB absorption spectrum. The PDI studies were performed with a red light and irradiation of the yeasts for up to five minutes. The authors observed an increase in the PDI effect of the AgNPs-MB when compared to bare MB. Complete inactivation of *C. albicans* could be achieved only after 2 min using 100 µmol.L^−1^ of the photosensitizer (AgNPs-MB). Moreover, the dosage of 50 µmol.L^−1^ associated with 4 min of irradiation was efficient in fully inactivating these fungi [131].

**Figure 9:**
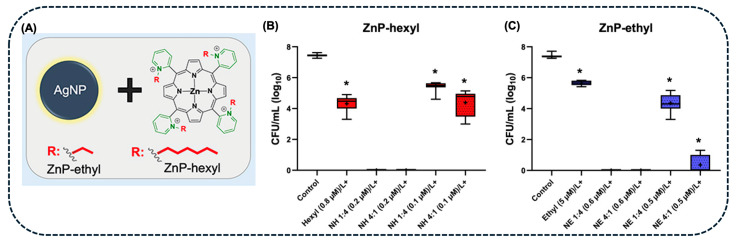
(**A**) Schematic representation of the association of AgNPs with zinc porphyrins (ZnP). PDI of *C. albicans* in the presence of systems containing (**B**) ZnP–hexyl and (**C**) ZnP–ethyl, with an irradiation time of 10 min under blue light. Hexyl: ZnP–hexyl; NH: AgNPs–ZnP–hexyl; L+: with light; Ethyl: ZnP–ethyl; NE: AgNPs–ZnP–ethyl. * *p* < 0.05 comparing to control. Reprinted from Raposo et al. [130], Copyright (2023), with permission from Dove Medical Press.

Porphyrins are very effective PSs for PDT. Thus, Raposo et al. [130] linked PVP-coated AgNPs with two zinc porphyrins (ZnP-ethyl and ZnP-hexyl) to photoinactivate *C. albicans* (Figure 9A). AgNPs-PVP were obtained with a spherical shape and a plasmon band at 413 nm. Both AgNPs-ZnP conjugates showed the absorption band from AgNPs, and two emission bands in the red region, corresponding to the presence of ZnP (Figure 9). The PDI studies showed complete eradication of *C. albicans* using a concentration of 0.2 μmol.L^−1^ of AgNPs-PS–hexyl or 0.6 μmol.L^−1^ of AgNPs–PS–ethyl, for 10 min under irradiation with blue LEDs (410 nm, 4.3 J.cm^−2^) (Figure 9B,C). This study showed that the association of the AgNPs with ZnPs enhanced the PDI efficiency of the PSs, allowing use of a lower dose [130].

### 3.6. Other Phototherapies Using AgNPs

AgNPs have also been associated with light to treat other diseases, such as neurodegenerative disorders [132] and benign prostatic hyperplasia [133], and as drug delivery systems.

Amyloid beta (Aβ) fibrils have been associated with Alzheimer’s and other neurodegenerative diseases. To dissolve mature Aβ plaques, Sudhakar et al. [132] used triangular AgNPs under NIR irradiation. The triangular AgNPs coated with PVP presented two absorption bands at 400 and 800 nm, and a size of around 70 nm. To study the dissolution of mature Aβ fibrils, the authors used Congo red dye, since its absorption at 540 nm is proportional to the fibrils. The NIR irradiation treatment did not affect the fibrils. However, when Aβ fibrils were incubated with AgNPs-PVP (30 nmol.L^−1^) and irradiated with NIR (800 nm, 200 mW.mm^−2^, 1 min), an almost complete dissolution of the mature fibrils was observed within 1 h. These results were confirmed by TEM images [132].

Benign prostatic hyperplasia (BPH) causes the enlargement of the prostate gland, by the accumulation of a huge quantity of stromal and epithelial cells. To treat these non-cancerous nodules, Marghani et al. [133] evaluated the use of spherical AgNPs-PVP (50–90 nm) as PTAs under light irradiation (532 nm, 1 W.cm^−2^), in rat models. After PTT treatment with a AgNPs-PVP dose of 50 mg.kg^−1^ for 5 min, the prostate size of the BPH-bearing animals was reduced compared with non-treated rats, showing that AgNPs could prevent the progression of BHP.

The development of drug carriers with controlled release has been growing, especially for poorly hydrophilic drugs. Plasmonic NPs (like AgNPs) convert a part of the energy received by light in heat, which is interesting for light-triggered drug delivery systems. With this in mind, Neri et al. [134] prepared AgNPs coated with poly(methacrylic acid) (PMA) and loaded them with sorafenib tosylate (SFT), a drug that inhibits tyrosinase protein kinases. The AgNPs-PMA-SFT capsules were obtained as a porous structure, with a diameter of 1 μm and pores of around 5–25 nm. These capsules showed a good drug release when activated by light irradiation, unlike the system without AgNPs. However, the drug release profile was dependent on the laser wavelength and power. The 420 nm laser showed faster release kinetics compared to the 620 nm laser. A 25 and 20% SFT release was observed with the laser of 420 nm and the power of 80 and 20 mW.cm^−2^, respectively, while, for the same period, the 620 nm laser achieved a 10% drug release. Despite the promising results, the authors reinforce the need for further studies [134].

## 4. Final Remarks

Over the last few years, AgNPs have been increasingly explored in phototherapy, reflecting the growing number of publications and the demand for more effective treatments. This review highlighted the latest advancements in the application of AgNPs across various light-based therapies. The main advantages of these treatment methods compared to traditional approaches, as well as the benefits of using NPs, include their minimally invasive nature, reduced side effects, and the small size of AgNPs, which facilitates interaction with diverse biological systems. Furthermore, their surface can be easily modified, enhancing specificity and applicability.

Direct comparison between the therapeutic tools reported, to define the most promising system, is not an easy task. The AgNPs’ dosage determination is complex and was not always determined in the same way; some researchers presented the concentration of Ag^+^, while others estimated the nanostructure mass per volume. Thus, it would be beneficial for the definition of a standard method to indicate the concentration, like the concentration in terms of Ag content, since it is easier to quantify the silver atoms/ions than the nanoparticles.

In the phototherapy experiments (PTT and PDT), key experimental parameters, such as AgNP concentration, laser wavelength, and power, often differ between reports. Hence, a common parameter needs to be established to allow the direct comparison between works and to understand what factors have a greater affect on the phototherapeutic efficacy of AgNP-based systems. For instance, the influence of AgNPs’ size, shape, and surface ligands on their therapeutic activity remains poorly understood. While the synthesis strategy appears to have minimal impact on their efficiency, these physiochemical features play a crucial role. Thus, studies focusing on the relationship between these parameters and AgNPs’ phototherapy efficiency are essential to enhance the therapeutic outcomes and support their successful clinical translation. Moreover, in the case of PTT experiments, the authors should consistently report the photothermal conversion efficiency (η), which can be readily calculated by the temperature raising curves, affording a universal parameter to evaluate and compare the performance of different AgNP formulations under varying experimental conditions.

An issue that still raises concerns with the use of AgNPs for therapy is their long-term biosafety. While existing *in vivo* studies indicate that AgNPs are biocompatible for normal cells, being selectively toxic to cancer cells and microorganisms, comprehensive long-term evaluations (more than one month) are still lacking. These studies are crucial to understanding the potential human health implications of prolonged exposure. Moreover, few studies have investigated the fate of the AgNPs after phototherapy. It is essential to determine whether AgNPs are excreted, how long they remain in the body, whether they accumulate in specific organs, or if they degrade and release Ag^+^ over time. Nevertheless, AgNPs’ potential for phototherapy is indisputable, and further works should focus on these issues and demonstrate their long-term consequences more carefully.

## Data Availability

No new data were created or analyzed in this study.
